# D-Sorbitol Physical Properties Effects on Filaments Used by 3D Printing Process for Personalized Medicine

**DOI:** 10.3390/molecules26103000

**Published:** 2021-05-18

**Authors:** Stéphane Roulon, Ian Soulairol, Maxime Cazes, Léna Lemierre, Nicolas Payre, Laurent Delbreilh, Jean Alié

**Affiliations:** 1 Group of Materials Physics, UNIROUEN Normandie, INSA Rouen, CNRS, Normandie University, Av. Université, 76801 St Etienne du Rouvray, France; 2 Solid State Characterization and 3D Printing Laboratory, SMO-A Department, Sanofi R&D, 371 rue du Pr. Joseph Blayac, 34080 Montpellier, France; Maxime.Cazes@sanofi.com (M.C.); Lena.Lemierre@Sanofi.com (L.L.); Nicolas.Payre@Sanofi.com (N.P.); 3 Department of Pharmacy, Nîmes University Hospital, 30900 Nimes, France; ian.soulairol@umontpellier.fr; 4 ICGM, University Montpellier, CNRS, ENSCM, 34000 Montpellier, France

**Keywords:** 3D printing, fused filament fabrication, hot melt extrusion, oral dosage forms, filament, personalized medicine, D-Sorbitol

## Abstract

Fused filament fabrication (FFF) is a process used to manufacture oral forms adapted to the needs of patients. Polyethylene oxide (PEO) filaments were produced by hot melt extrusion (HME) to obtain a filament suitable for the production of amiodarone hydrochloride oral forms by FFF 3D printing. In order to produce personalized oral forms adapted to the patient characteristics, filaments used by FFF must be controlled in terms of mass homogeneity along filament. This work highlights the relation between filament mass homogeneity and its diameter. This is why the impact of filler excipients physical properties was studied. It has been showed that the particle’s size distribution of the filler can modify the filament diameter variability which has had an impact on the mass of oral forms produced by FFF. Through this work it was shown that D-Sorbitol from Carlo Erba allows to obtain a diameter variability of less than 2% due to its unique particle’s size distribution. Using the filament produced by HME and an innovating calibration method based on the filament length, it has been possible to carry out three dosages of 125 mg, 750 mg and 1000 mg by 3D printing with acceptable mass uniformity.

## 1. Introduction

One of the great challenges of the 21st century in the pharmaceutical field is to offer drugs with a dosage adapted to the physiological needs of each patient. Studies show that more than 100,000 patients hospitalized in the United States die each year because of a reaction to the active ingredients. In addition, more than 2 million patients develop serious reactions to drugs requiring hospitalization [1]. Between 75% and 80% of undesirable effects are linked to the dose of active ingredient, which means that the prescribed dose was in certain cases unsuitable for the patient [2]. The modification of the metabolism in the elderly can modify the absorption of the active ingredient while in children, the variation in weight between individuals is such that the need for the active ingredient can vary by a factor of 20 [3]. This context highlights the need to adapt the dose of active ingredient according to the patient’s characteristics. Three-dimensional printing technology can adjust the amount of active pharmaceutical ingredient (API) in oral forms as needed. This would make it possible to reach a milestone in the field of personalized medicine.

Three-dimensional printing includes a variety of techniques such as selective laser sintering, stereolithography, gel and fused filament fabrication [4]. FFF 3D printers are small machines which are used in the field of aerospace, prostheses and in oral forms production [5,6]. This technology uses a filament as raw material which can be made of thermoplastic polymer loaded with the active ingredient in an extrusion head [7]. The molten filament is deposited on a support and then solidifies. The next layer is added to the previous solidified layer until the desired size and shape are obtained. This is called additive manufacturing. This technology allows to directly print the drugs closest to the patient (hospital or city pharmacies for example) while adapting the dose to the patient’s weight [8,9]. Three-dimensional printers can also be used in clinical study centers to adjust the dose according to the need [10]. It is in this context that Merck has partnered with a manufacturer of 3D printers (EOS) to produce next-generation 3D printed tablets [11]. Moreover, many teams have worked on the formulation of oral forms by 3D printing for immediate release [12], delayed release [13,14] and the combination of immediate and delayed release within an oral form [15]. To produce immediate release formulations by FFF 3D printing, thermoplastic polymers selected are generally Eudragit^®^ EPO, Kollicoat^®^ IR, PEO, Kollidon^®^ VA64, PVP and PEG [6].

In accordance with Official Journal of the European Union guidelines of 19 March 2015 (with European economic area relevance), the formalized risk assessment for ascertaining the appropriate good manufacturing practice for excipients of medicinal products for human use show that the excipient risk assessment should be incorporated in the pharmaceutical quality system of the manufacturing authorization holder. Three-dimensional printing is a new way to produce medicines. This is why excipients’ impact on the critical quality attributes of the medicinal product have been studied in this publication as requested in chapter 2, part 4 of the Official Journal of the European Union guidelines [16].

Filament diameter manufactured by HME varies along the length of the filament due to variations in flow rate at the extrusion outlet. Filament diameter determines the quantity of material over a given length. The 3D printing process makes an object with a given length of filament. Therefore, variations in diameters result in mass variability of oral forms fabricated by FFF 3D printing. Process plays an essential role in the reproducibility of the filament diameter. Indeed, it has been shown that the extruder and screw choice is important to have a homogenous mixing [17]. It has also been demonstrated that large variation in particle size between the polymer and the drug reduces the homogeneity of dispersion of the active principle in the extruded filament [18]. Many studies concerning the HME process of pharmaceutical filaments have focused on the homogeneity of drug dispersion [19]. This is why the work presented here will focus on variations in filament properties such as diameter and mass for a given length.

Three-dimensional printed formulations consist of different products. Thermoplastic polymers are a class of products used to produce 3D-printed medicines by its ability to melt and solidify in a reversible way. Properties’ impact of this type of product on medicinal products are widely studied [20,21,22,23,24,25], including as plasticizers [26,27,28,29,30]. The polymer allowing the manufacture of the filaments in this work is polyethylene oxide (PEO). PEO is a water soluble polymer [31] which is known to be printable by FFF process [22]. PEO provides good mechanical flexibility to the filament and is extrudable at temperature lower than 100 °C [32]. Glycerol has been selected for these plasticizing properties [33,34,35] in order to reduce the extrusion temperature and obtain a flexible filament that can be used by the FFF 3D printing process [27,36]. Colloidal anhydrous silica is used to obtain flow properties allowing an even feed of the powder into the extruder [37,38].

The drug used in this study is amiodarone hydrochloride for pediatric use. Amiodarone hydrochloride is an antiarrhythmic drug used to treat patients with arrhythmias [39]. Some hospitals are currently having to prepare oral forms to adjust the dosage of amiodarone delivered. In fact, the use of medicines in children requires dosage adjustment according to the patient characteristics (weight, body surface…). This is why the development of 3D-printed oral forms containing amiodarone hydrochloride can make it possible to produce oral forms on demand and in a personalized way.

One of the products used in the largest quantity was the filler agent. It allowed the reduction of the amount of thermoplastic polymer used in order to obtain better dissolution kinetics [25]. Few studies reported the use of filler agent to produce medicines by FFF 3D printing [23]. In this work, the filler chosen to produce a fast disintegration formulation is D-Sorbitol. D-Sorbitol is a filler with a high solubility [40]. This product has been used in 3D printing formulation as plasticizer in combination with PVA [30] or as a temporary plasticizer [29]. A supplier change can influence physical properties of an excipient. In this work, 37% (in mass) D-Sorbitol was used. Therefore, a supplier change can have a big impact on filament and oral form quality [22]. D-Sorbitol origin is therefore important in order to choose the most adapted supplier to obtain most suitable oral form quality.

In the present work, D-Sorbitol from different suppliers are characterized with the aim to compare their morphology, size and flowability properties. All D-Sorbitol were incorporated in a formulation to produce filaments by HME technology. Their impact on processability and filament properties were analyzed. Filament batches were used to produce 3D-printed medicines. The influence of filament properties such as diameter, mechanical properties and apparent density on oral form quality was evaluated.

## 2. Materials and Methods

### 2.1. Materials

D-Sorbitol Parteck SI150 (SI150) and Parteck SI200 (SI200) were donated from Merck, Darmstadt, Germany. D-Sorbitol (CE) was purchased from Carlo Erba, Italy and D-Sorbitol (GLT) was purchased from Glentham, Corsham, UK. Polyethylene oxide, Polyox N10 (PEO) was donated from Dow Chemical, Midland, TX, USA. Glycerol was purchased from VWR, Radnor, PA, USA. Colloidal anhydrous silica (Aerosil 200 Pharma) was purchased from Evonik, Rheinfelden, Germany, USA. Amiodarone hydrochloride was donated from Sanofi, France.

### 2.2. Methods

#### Blending

First, the thermoplastic polymer, PEO was weighed then the liquid plasticizer (glycerol) was added by mixing/grinding in a mortar. Once the preblend was homogeneous, the active ingredient (amiodarone hydrochloride) was added to the previous mixture by mixing/grinding. The same process was used to add the filling (D-Sorbitol) and the glidant agent (colloidal anhydrous silica).

The excipients were chosen for the reasons described in a previous publication [41].

CE D-Sorbitol presented a large quantity of small particles. To control impact of this particles on the process, a 45-micron sieve (Test Sieve, Haver & Boecker, Oelde, Germany) was used. The fraction of D-Sorbitol greater than 45-microns was used under the name CE^T^.

The powder formulations are summarized in Table 1. All batches were formulated with the same products ratio to obtain a reproducible formulation dosage. All batches were dried at least 12 h at 20 °C in the Fisher Scientific Bio Block oven (Fisher Scientific, Waltham, MA, USA) (with 900 mbar vacuum and silica gel in the oven). Some of our results regarding D-Sorbitol CE and formulation A were presented in a previous work [41].

Formulations presented in Table 2 contains an identical ratio of each component presented above. However, the origin of the D-Sorbitol tested varies depending on the batches manufactured. Formulation A and A^T^ contained respectively nonsieved and sieved Carlo Erba D-Sorbitol, formulation B and C contained Merck D-Sorbitol and formulation D contained Glentham D-Sorbitol. All other products contained in formulations were identical in terms of quality and quantity.

### 2.3. Hot Melt Extrusion (HME)

The formulated powder was added in the force feeder (Thermo Fisher Scientific, Karlsruhe, Germany) which is a volumetric feeder at a screw speed of 3.5 RPM. This allows the powder to be drawn into the extruder. HME was carried out using a Pharma Mini HME with conical corotating twin screw extruder at a speed of 25 RPM and at an extrusion temperature of 50 °C and 80 °C on the first and second half of the extruder, respectively with an aluminum rod-shaped die (Ø = 1.75 mm) (Thermo Fisher Scientific, Karlsruhe, Germany). The temperature of the laboratory environment is 20 °C.

The filament diameter were controlled by a stericut-1T (Citius engineering, Ougrée, Belgium) which carries out a regulation loop. Depending on the physical quality and the constituents of the formulation, the fluctuation around this target diameter will be more or less important. It was therefore necessary to fully understand formulation properties that modify filament diameter. Diameter was adjusted with 1.70 mm by belt speed adjustment.

### 2.4. Mechanical Testing

Filaments mechanical properties were tested on a texture Analyzer TA-XT plus (Stable Micro Systems, UK) equipped with a three-point bend rig HDP/3PB (Stable Micro Systems, UK) [42]. Experiments were conducted with a blade speed of 3 mm/sec and a total displacement of 10 mm. The triggering force of the analysis was fixed at 0.25 N in order to limit the influence of weak forces which can trigger the analysis at different distances depending on the sample orientation. Support spacing was 25 mm and filaments were attached on either side of the support to allow easier reading of deformation profile and better results reproducibility. Tests were done in triplicate for all tested filaments. Three Filaments samples were cut to a length of 4 cm. Exponent version 4,0,13,0 software (stable micro Systems, UK) was used for data analysis and recovery.

### 2.5. Thermal Analysis

Samples of raw materials, filaments and oral forms were characterized using Differential Scanning Calorimeter DSC Q2000 (TA instruments, Elstree, Hertfordshire, UK) with a heating rate of 10 °C/min [43]. Samples were heated with a single ramp from 20 °C to 190 °C. Analysis were carried out under a purge of nitrogen (50 mL/min). D-Sorbitol peaks were integrated from 75 °C to 115 °C using TA 2000 universal analysis software (TA instruments, Hertfordshire, UK). Standard 40 µL TA aluminum pans and lids were used with an approximate sample mass of 5 mg.

For thermogravimetric analysis (TGA), raw materials, filaments and 3D printed tablets were analyzed using a TGA Q500 (TA instruments, Hertfordshire, UK). Samples (10 mg) were placed in 40 µL aluminum pans and were scanned from ambient temperature to 300 °C at a heating rate of 10 °C/min. The weight loss profile was studied using TA 2000 universal analysis software (TA instruments, Hertfordshire, UK). Experiments were carried out under a nitrogen gas flow of 40 mL/min and 60 mL/min for sample and furnace respectively.

### 2.6. X-ray Powder Diffraction (XRPD)

The powder X-ray diffraction analysis was carried out by a D8-Discover Bruker diffractometer with a copper anticathode tube with Kα radiation (λ = 1.540562 A) and Ni filter with a thickness of 0.5 mm. The voltage and current of the tube used are 40 KV and 40 mA respectively. Samples were scanned over a 2-theta angle range from 2° to 40° with a step of 0.03° and a time per step of 0.5 s. The study of diffraction patterns was carried out using EVA version 5.1.0.5 software.

### 2.7. Particle Size Analysis Dry Dispersion Method (PSD)

Powder particle size was measured by laser diffraction (Mastersizer 3000, Malvern, GB) coupled with a dry dispersion unit without additional pressure in order to observe PSD repartition of particles in the product.

### 2.8. Scanning Electron Microscopy (SEM)

In order to assess the distribution and morphology of the various components in the mixtures, analysis was carried out using a scanning electron microscope (SEM) (JSM-IT 500HR, Jeol, JP). Before any observation, samples were placed on adhesive carbon tabs, themselves fixed on an aluminum specimen holder.

### 2.9. Flowability Evaluation

Powder flow properties of all D-Sorbitol have been evaluated by various techniques. The angle of repose was measured with a 10 mm diameter stainless steel funnel (Flowability tester BEP2, Copley Scientific, UK). The height between the end of the funnel and the support was 75 mm. The diameter of the support was 25 mm.

**Figure 1:**
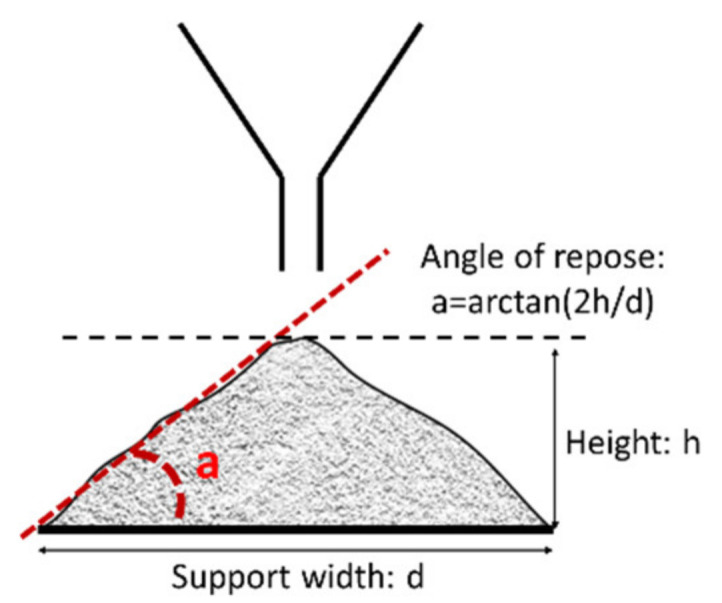
Scheme of angle of repose measurement of powder.

After the formation of a powder pile by saturation of the support as shown in Figure 1 the height of the cone (*h*) is measured and the angle of repose was calculated using Equation (1):

(1) $$\theta =\tan^{-1}\left(\frac{2h}{d}\right)$$

The diameter (*d*) corresponds to the support used (here 25 mm). All the samples were analyzed three times to obtain a better representativeness of the results.

Bulk density study was also carried out (TAP density USP1 TD2, Sotax, SWI). This experiment allowed the calculation of Hausner ratio and Carr’s index according to European pharmacopeia [44,45]. There are indicators of powder flowability and can be calculated using Equations (2) and (3):

(2) $$Carr\;index=100*\left(V_{10}-V_{500}\right)/V_{10}$$

(3) $$Hausner\;Ratio=V_{10}/V_{f}$$

*V*_10_ corresponds to the volume occupied by the powder after 10 taps. *V*_500_ corresponds to the volume occupied by the powder after 500 taps. *V_f_* corresponds to the final volume of last test sample.

### 2.10. Three-Dimensional Printing of the Dosage Forms

A cube of 10 mm side was modeled using Openscad software version 2015.03. The design was then imported to the 3D printer’s software Repetier version 2.1.6.

FFF 3D printing were performed using a Prusa i3 Mk3S printer equipped with a 0.6 mm nozzle (Prusa Research, Prague, Czech Republic). Oral forms were printed on a steel tray with a smooth polyethyleneimine coating supplied with the printer. Oral forms were printed using the settings presented in Table 3. The nozzle temperature was optimized to obtain a constant flow as well as oral forms with suitable resolution.

Once the slicing operation is performed, the length of filament to be used by the printer is indicated by the printer driver software. Three filaments lengths (50.2, 301.2 and 401.6 mm) are imposed by modifying the dimensions of the object directly in the slicing software to produce three oral forms by filament length tested for all formulations.

To avoid any cross-contamination, the 3D printer was dedicated throughout the duration of the study to the manufacture of amiodarone hydrochloride oral forms.

### 2.11. Disintegration of Oral Forms in Syringe

The disintegration time of the oral forms was tested under conditions similar to ones done in hospitals. Therefore, each experiment was carried out on an oral form which was put in a syringe containing 5 mL of water. The plunger was then added to the syringe and manual agitation was performed. The complete disintegration time was recorded. Experiments were carried out in triplicate by the same manipulator.

## 3. Results and Discussion

### 3.1. Characterization of D-Sorbitol

**Figure 2:**
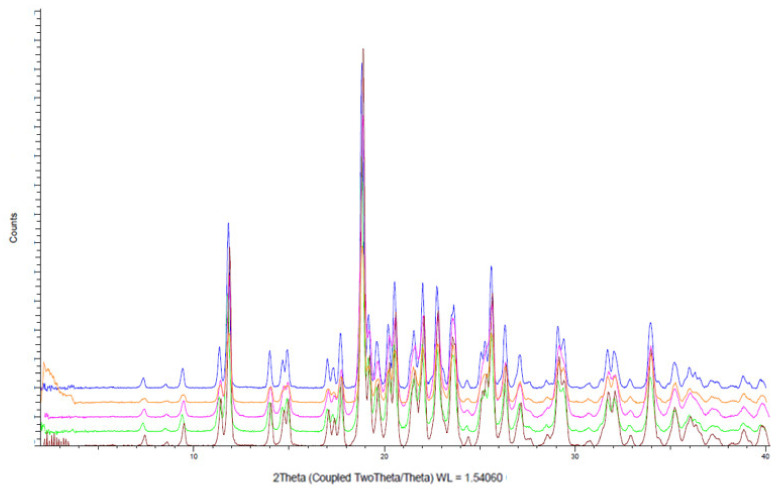
X-ray powder diffraction analysis of D-Sorbitol GLT (**blue**), CE^T^ (**yellow**), CE (**red**) [41], SI150 (**green**) and SI200 (**pink**).

Polymorphism tendency is known to be an essential data for the development of an oral form. A modification of D-Sorbitol crystalline form can impact flowability of the entire formulation. [46]. Therefore, all the D-Sorbitols selected for this study were analyzed by X-ray powder diffraction in order to verify their crystalline form. The five D-Sorbitols present an identical XRD pattern that proves their existence in the same crystalline form as showed by Figure 2.

In order to observe the impact of the size and shape of the particles on the entire formulation extrusion process, five D-Sorbitol with different particle properties were selected.

**Figure 3:**
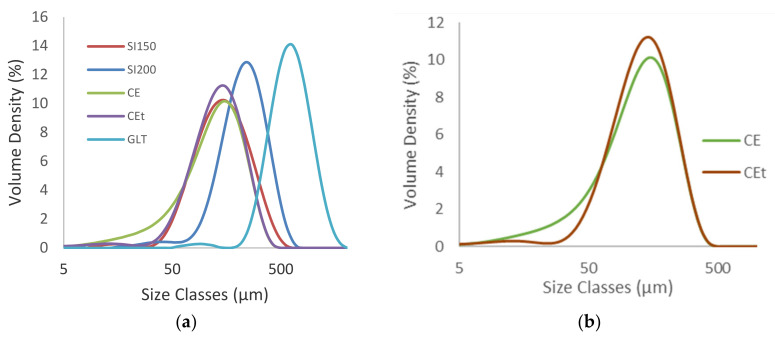
PSD comparison of D-Sorbitol CE, CE^T^, SI150, SI200 and GLT powders (**a**) and focus on CE D-Sorbitol sieved (red) and nonsieved (green) (**b**).

PSD measurement of D-Sorbitol Figure 3 highlight a monomodal particles distribution. CE and SI 150 have the lowest particles diameters with a D_v_^50^ of 125 µm and 142 µm respectively. SI 200 have a D_v_^50^ of 228 µm. GLT have the highest particles size with a D_v_^50^ of 606 µm. CE D-Sorbitol presented a significant quantity of particles smaller than 50 µm in comparison to others D-Sorbitol even though the observation of the diameter in volume minimizes the presence of small particles. Sieved D-Sorbitol CE^T^ no longer has particles smaller than 45 µm which proves the effectiveness of the sieving process. This D-Sorbitol therefore presents higher D_v_^50^ at 132 µm.

**Figure 4:**
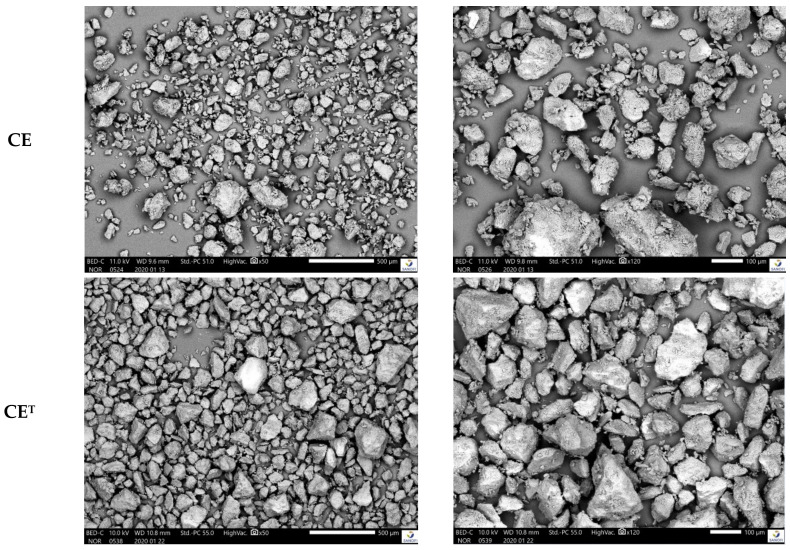

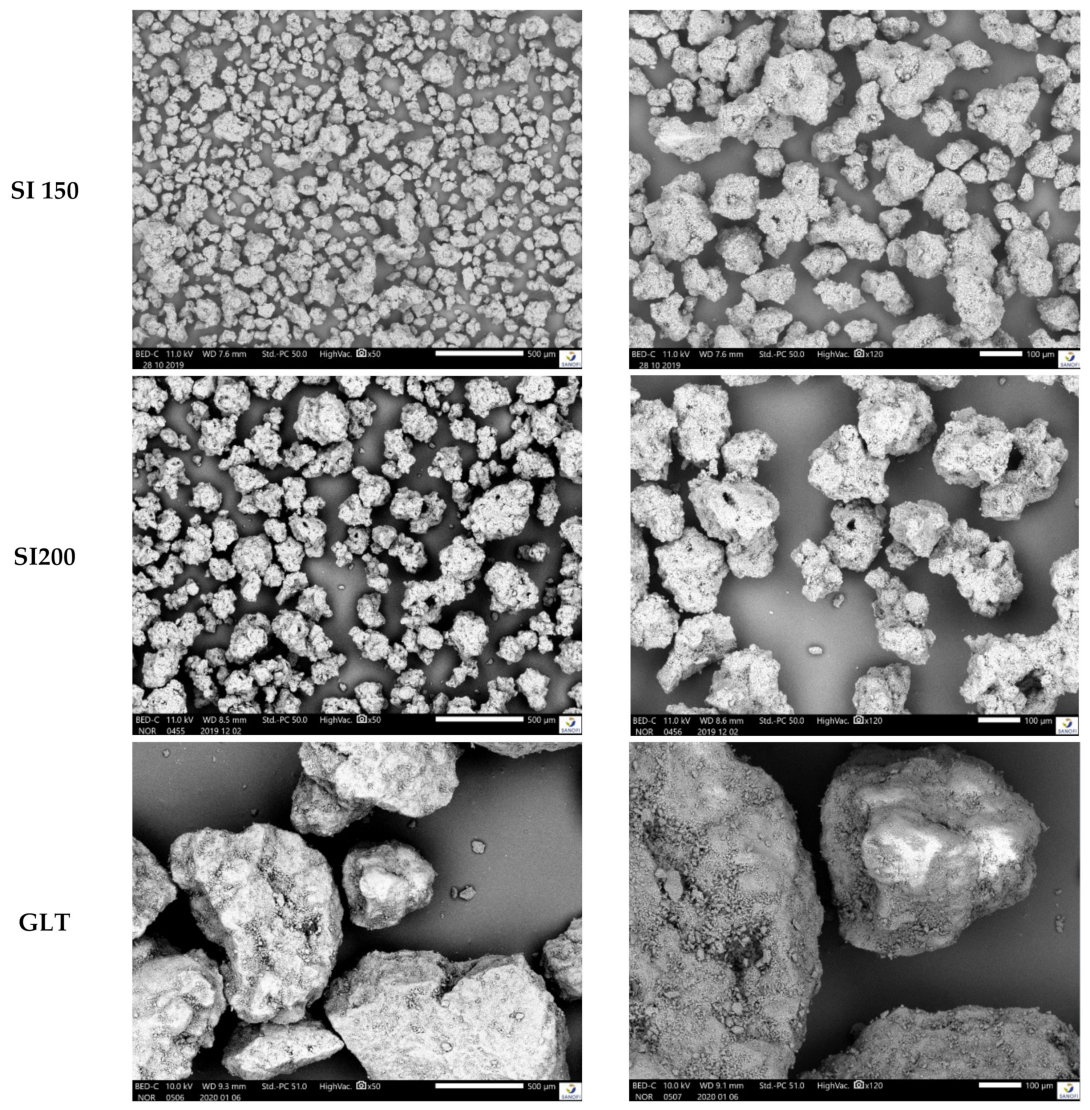
SEM pictures of D-Sorbitol from top to bottom CE, CE^T^, SI150, SI200 and GLT at X50 magnification (**top**) and X120 magnification (**bottom**).

D-Sorbitol presented similar particle morphology as presented by SEM pictures in Figure 4. Nevertheless, D-Sorbitol SI 150 and SI 200 had an identical particles morphology unlike other D-Sorbitol which were different in terms of particle morphology. The influence of D-Sorbitol CE sieving was highlighted by the SEM images. Indeed, CE presented particles smaller than 50 microns in contrary to CE^T^.

**Figure 5:**
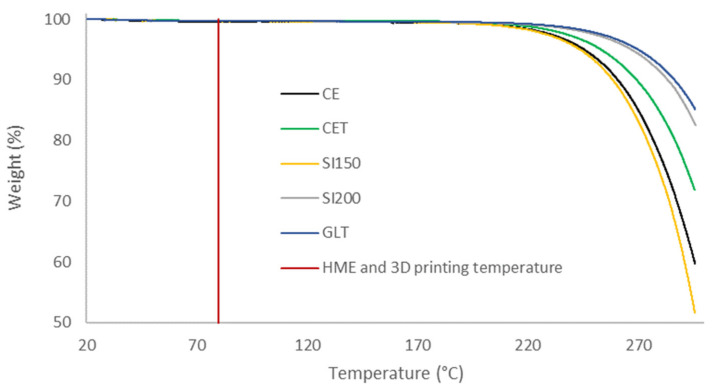
TGA thermal decomposition profile of D-Sorbitol CE, CE^T^, SI150, SI200 and GLT powders [41].

Powder moisture can impact formulation flowability and product quality [47,48]. TGA was carried out to observe difference in mass loss between 20 °C and 115 °C corresponding to water loss. TGA graphs in Figure 5 showed that below degradation temperature, the mass losses were similar, and no link were observed between the particle characteristics and the water losses between room temperature and 115 °C (Table 4). D-Sorbitol showed a degradation starting at 200 °C. The HME and 3D printing process imposed a temperature of 80 °C on the components of the formulation. No degradation of D-Sorbitol was expected to occur during HME and 3D printing.

Particles shape and size are well known to be melting onset modifier [49]. For organic compounds, it has been showed that the melting point lowers as the particle size decreases [50]. Melting temperature is one of the characteristics which can influence HME settings. Therefore, it is important to assess the impact of the filling agent physical quality on the melting point.

**Figure 6:**
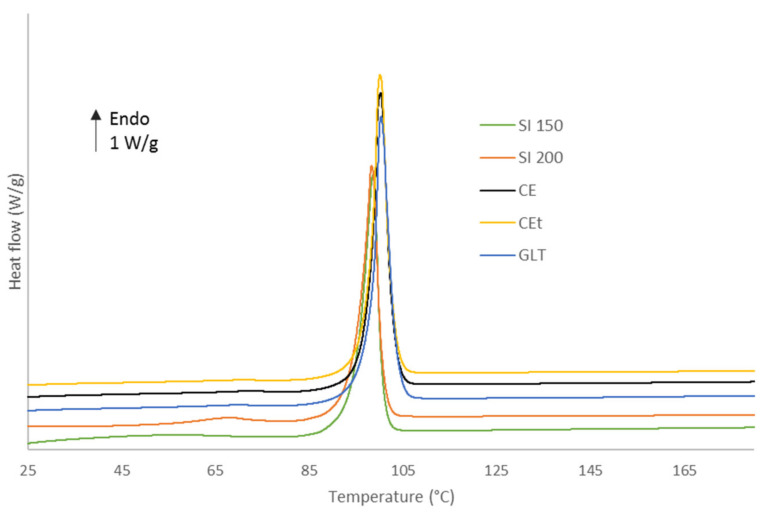
DSC thermoanalytical curves of D-Sorbitol CE [41], CE^T^, SI150, SI200 and GLT powders.

D-Sorbitol melting onsets are observed in thermoanalytical curves presented in Figure 6. Two groups in terms of melting onset were observed. The first group was formed by CE, CE^T^ and GLT. These powders presented a melting onset around 98 °C. The second group was formed by SI 150 and SI 200. These powders presented a melting onset around 96 °C (Table 4). Each group seemed to present a similar particle’s shape indicating that it may played a role in the melting onset value contrary to the particles size which was not sufficiently far from a supplier to the other to have an influence on the melting onset.

Each of the D-Sorbitol used in the formulation had different particle sizes and shapes. These characteristics induce changes in flow properties [51]. Flowability was an important bulk powder characteristic. Angle of repose, Carr’s index, Hausner ratio were used to express powders flowability [51,52].

It has been reported by Geldart et al., that angles of repose below 30° reflect a good flowability, for angles of 30–45° some cohesiveness, 45–55° true cohesiveness and >55° very high cohesiveness and very limited flowability [52]. A Hausner ratio below 1.18 and a Carr’s index below 15 correspond to a good to excellent powder flow properties [53].

It is well known that particle size and distribution are properties that influence powder flow [54]. As observed Table 5, CE D-Sorbitol present high cohesiveness compared to others D-Sorbitol which is highlighted by a high Carr’s index of 24.2 and an angle of repose of 30.2°. All powder flow indicators allow to conclude that CE is the most cohesive D-Sorbitol. Reducing particles size increases surface area and so surface cohesive forces which induce interactions and resulting in a more cohesive flow behavior [55,56,57]. This phenomenon explained the difference of flowability between CE and CE^T^ as reflected by a Carr’s index of 24.2 for CE D-Sorbitol and 22.0 for CE^T^ D-Sorbitol. The particle sizes of D-Sorbitol SI 150, SI 200 and GLT were more important than those of D-Sorbitol CE and CE^T^ as showed previously which explained why their flow indicators reflected a better flowability.

### 3.2. Filaments Production and Characterization

**Figure 7:**
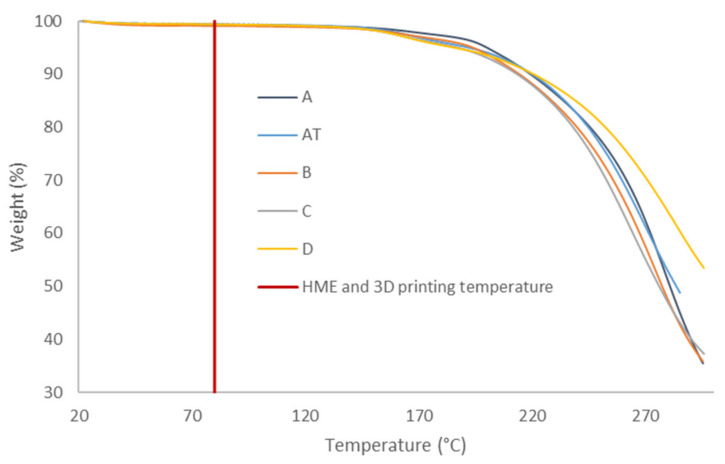
TGA thermal decomposition profile of formulations A, A^T^, B, C and D in state of powders [41].

In order to compare the thermal decomposition pattern of the formulations in state of powders, thermogravimetric analyses were carried out (Figure 7). Samples’ weight loss revealed that all formulations do not contain significant amounts of water because of the 1% mass loss after reaching 115 °C. The degradation starts at 160 °C due to amiodarone hydrochloride degradation. Therefore, no degradation has been expected during HME and 3D printing because of a process temperature of 80 °C.

**Figure 8:**
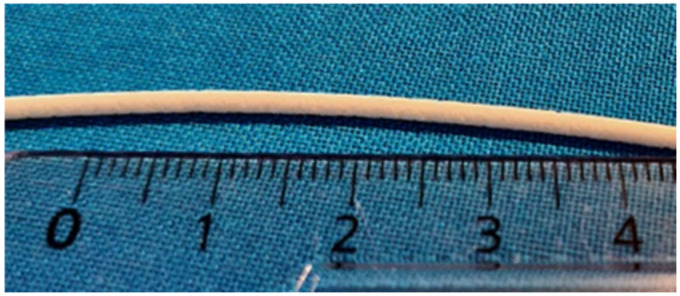
Picture of filament produced by HME (the five formulations have the same aspect).

HME process gives white and opaque filaments due to the large quantity of crystalline products as shown in Figure 8.

Five powder mixtures were tested in the extrusion process to produce pharmaceutical filaments. Depending on the formulation used, filament diameter measured by stericut vary. When a material comes out of the extrusion nozzle, the stericut measures its diameter and adjusts the belt speed to compensate any variations in flow. It has been identified that the delay between the diameter measurement and belt speed regulation induces a diameter variability. Moreover, a larger variability was observed when the flow presented significant oscillations. A filament diameter variability led to a mass variability which was of the same order of magnitude on the filament mass and 3D printed oral forms. Indeed, a filament with a larger diameter will contain, for a given length, more material.

All the D-Sorbitol powder flow indicators Table 5 permitted us to conclude that D-Sorbitol CE present some cohesiveness. This product provided a formulation with a filament diameter standard deviation of 1.5% which was the best quality of filament obtained. Other D-Sorbitol gave a filament diameter variability of more than 2.0% but presented better powder flowability results. However, the study conducted on the formulated powders angle of repose did not make it possible to highlight significant differences in flow on the formulation (data not shown). Formulations’ flowabilities were equivalent, which was confirmed by an equivalent speed of regulation between 6.11 mm/s and 6.67 mm/s Table 6. Stericut regulation speed were directly correlated to the flow rate of the powder entering the extruder and this flow rate was linked (at constant force feeder speed) to the powder flow properties. The absolute flow property of the formulation was not the parameter influencing standard deviation in all formulation. On the contrary, the flow variability induced variability in the HME flow rate and as a result in the filament diameter. It was therefore important to consider the overall flow of the formulation but also the flow variability in the system. As the different grades of D-Sorbitol have different melting temperature and the HME process only melted part of the D-Sorbitol during HME, the viscosity of the mixture containing D-Sorbitol could be different. However, sieved (CE^T^) and unsieved (CE) D-Sorbitol have the same melting onset but do not exhibit the same variability in diameter (respectively 2% and 1.6%). D-Sorbitol GLT had the highest melting onset (98.4 °C) and SI 200 the lowest (95.7 °C), their coefficient of variation of diameter nevertheless remains close and respectively to 2.4% and 2.5%. This demonstrates that the thermal properties of D-Sorbitol are not the parameters modifying the RSD of filaments diameter.

D-Sorbitol particle size, morphology and filament diameter analysis allowed different observations. D-Sorbitol SI 150 and SI 200 had different particle sizes (D_v_^50^ of 142 µm and 228 µm respectively) while possessing equivalent particle morphologies. However, the variation in filament diameter standard deviation remains equivalent to 2.9% for formulation B and 2.5% for formulation C. This means that the change in particle size does not significantly influence the standard deviation. The same observation can be made by comparing the GLT and SI 200 filament diameter standard deviation which are equivalent and respectively equal to 2.4% and 2.5%. GLT D-Sorbitol particles have a D_v_^50^ of 606 µm in comparison to SI 200 that have a D_v_^50^ of 228 µm. The filament diameter variability is not modified by those differences. Therefore, a particle size variation beyond 45 microns does not seem to significantly modify the standard deviation of the filament produced by HME. However, D-Sorbitol CE sieving increase filament diameter variability. From 1.6% of filament diameter variability when not sieved D-Sorbitol is used (formulation A) to 2% of filament diameter variability when sieved D-Sorbitol is used (formulation A^T^). Particles smaller than 45 microns in CE D-Sorbitol seem to have a stabilizing effect on the flow rate of the powder entering the extruder.

**Figure 9:**
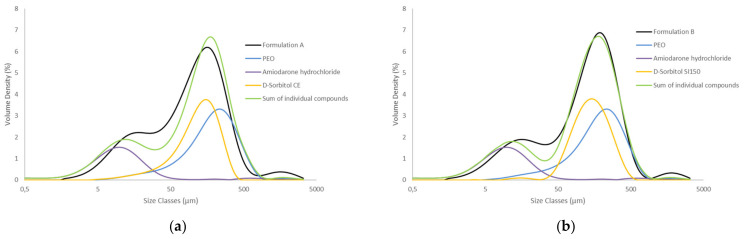
PSD comparison of individual components of the formulation, API, SI150, CE and PEO, the formulation A (**a**) and B (**b**) represented in black after mortar mixing and superposition of individual components.

The PSD graph in Figure 9 can explain impact of particles around 30 µm to obtain a constant powder flow. In fact, the PSD graph Figure 9b represents the particle size distribution of the products contained in the formulation B respectively with respect to their quantity by mass in the formulation. Colloidal anhydrous silica is present in small quantities in the formulation, which is why it is not visible in the graph. Glycerol is a liquid plasticizer added into the formulation and no PSD measurement has been done on this product. All compounds’ particle distributions are added to obtain the green curve representing the particle size state of the theorical mixture. This mixture has particles of API centered around 12 microns as well as particles of D-Sorbitol and PEO centered around 200 microns. A small number of agglomerated particles is present around 1700 microns. The particle size distribution of the mixed and ground formulation is shown in black. The curve is equivalent to the theoretical mixture showing that the addition of glycerol and the grinding does not granulate the powder and does not significantly modify particles size distribution. The formulation particles size distribution highlight that the two particles population are separated by a hollow around 35 µm. Therefore, formulation B has a small number of particles around 35 microns. A similar PSD graph is presented in Figure 9a with the formulation A, CE is the only D-Sorbitol to present a significant quantity of 35 µm particles. This could allow a particle size continuum which is supposed to induce a more constant flow by avoiding the formation of particle voids around 35 microns.

Formulated powder at the alimentation zone has been considered as the flow variability origin. However, it has been reported that formulation in state of melted product in HME can be the origin of some flow variability [57]. Indeed, for linear polymers as polyethylene oxide, oscillation of flow volume and extrusion pressures are frequently observed and known as stick slip melt fracture [58,59]. This oscillation phenomenon occurs at high shear rate and can be diminish by the addition of filler into the polymer matrix [60]. Filler size has been reported to influence the stick slip phenomena [61]. In our case, the extruder was equipped with a conical corotative twin-screw system which allowed a gradual increase in pressure along the screws [62]. This system allows a stable pressure during production. Moreover, flow rate and pressure are small in our hot melt extrusion system, so the shear rate is low. This oscillatory phenomenon that is sometimes present during polymer extrusion has therefore been ruled out.

Once the filament is produced, it can be used by 3D printer. During 3D printing process stream, the filament is subsequently fed into the FFF 3D printer. Inside the 3D printer, a feeding gear system drives the filament. Therefore, suitable mechanical properties of the filament are essential for the feeding process. The three-point bending test gives information about filament properties [63,64]. If it is brittle or too flexible, it cannot be used by 3D printing.

**Figure 10:**
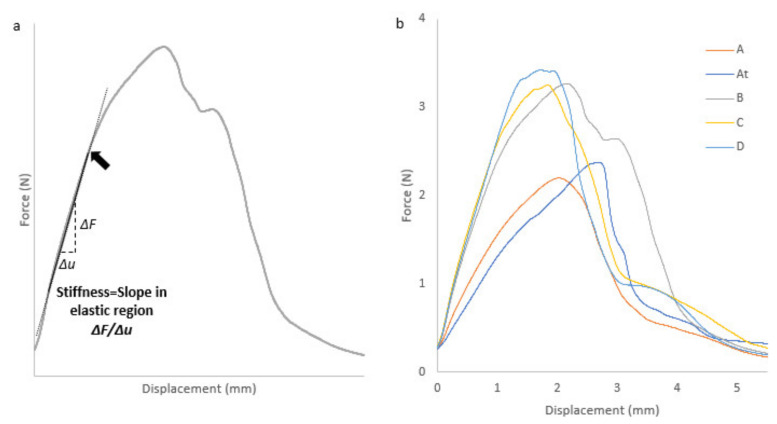
Stress–strain curve of filament sample in tensile tests. An arrow indicates the elastic limit (**a**). Stress-Strain curve of filaments corresponding to formulation A [41], A^T^, B, C, D (**b**).

In a curve representing the force as a function of the displacement (Figure 10), the first linear part of the curve represents an elastic behavior of the filament. The linear domain allows the obtention of filaments’ relative stiffness. When the curve is no longer linear, one passes from an elastic, and reversible strain to an irreversible strain. This transition is characterized by the elastic limit (pointed by the black arrow on Figure 10). The maximum stress on the curve corresponds to the breaking point of the filament.

Different teams performed three-point bending analysis on pharmaceutical filaments [26,64,65]. They used the three-point bending analysis without attaching the filament to the support. As the filament is a curved material that can be oriented differently depending on the sample, it was decided to attach it to two holding points. In addition, they used the point of maximum strength as a value characterizing filament property because of the difficulty to determine the elastic limit due to filament ductility. Given the large amount of nonmelted material contained in our formulation and the fact that the filament is attached to the support, it was decided to use the elastic domain limit as shown by the black arrow in Figure 10 (obtained without offset). The latter is easily observable (on our formulations) and represents the point where the filament can present nonelastic comportment.

All of the extruded formulations were analyzed (Figure 10) to obtain a force-distance curve. The samples all exhibit elastic then plastic behavior, ending with the breaking of the filaments. Extrudates exhibit ductile behavior represented by the appearance of plastic deformation in the force–distance curve.

Elastic distance is reported in Table 7 to be greater than 1 mm for all formulations. D-Sorbitol grade and origin does not impact elastic strain of the filament produced. Elastic domain size is therefore not really influenced by size and particle shape.

However, filament stiffness is a parameter modified depending on the D-Sorbitol origin. The slope in elastic region is directly correlated to filament stiffness properties. A steep slope indicates a high filament stiffness. As shown in Table 7, formulation B and C present an elastic domain slope of 2.0 N/mm and 2.1 N/mm which is equivalent. These formulations contain respectively SI 150 and SI 200 which are D-Sorbitol of identical morphology but different size. It shows that particle size in this case didn’t impact filament stiffness. The same conclusion can be done with A and A^T^ because they present different particles size and an equivalent stiffness of 1.2 N/mm and 1.0 N/mm. Filaments stiffness containing D-Sorbitol GLT (formulation D) is close to formulations B and C. However, D-Sorbitol GLT has a particle morphology closer to the D-Sorbitol used in formulations A and A^T^.

Shao-Yun Fu and al, shows that composite modulus (linked to stiffness) is generally independent of particle size. However, when the particle size is decreased to a critical size of 30 nm, particle size will present an obvious effect on the stiffness [66,67]. This explains the small impact of D-Sorbitol size on filament stiffness. Moreover, two groups can be identified in terms of stiffness. Formulation B, C and D present a stiffness around 2.1 N/mm and formulation A and A^T^ a stiffness around 1.1 N/mm. It has been reported that a large aspect ratio is generally considered as mechanical properties modifier [66]. It is supposed that particles morphology impact filament stiffness and should be considered when an unmelted filler is chosen.

Filaments were usable by 3D printing whatever the D-Sorbitol supplier. D-Sorbitol origin does not modify the elastic distance of the filaments produced as well as their macroscopic appearance. However, three-point bending analysis has showed through elastic domain slope that particle aspect seems to have an influence on filament stiffness.

### 3.3. Three-Dimensional Printing of Fast Disaggregation Oral Forms

**Figure 11:**
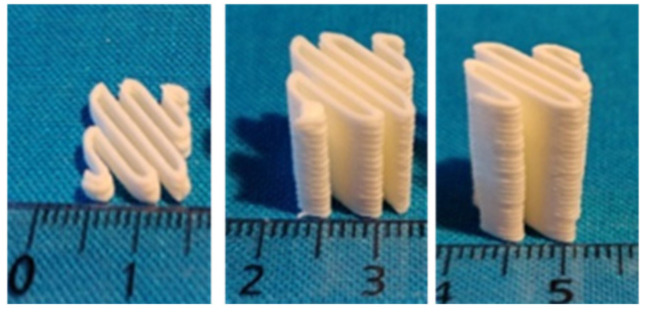
Three-dimensionally printed oral forms for dispersion in aqueous medium. From left to right: 125, 750 and 1000 mg.

Using filaments manufactured by HME, it is possible to produce oral forms with a dosage adapted to each patient by adjusting the size of the 3D printed object (Figure 11).

**Figure 12:**
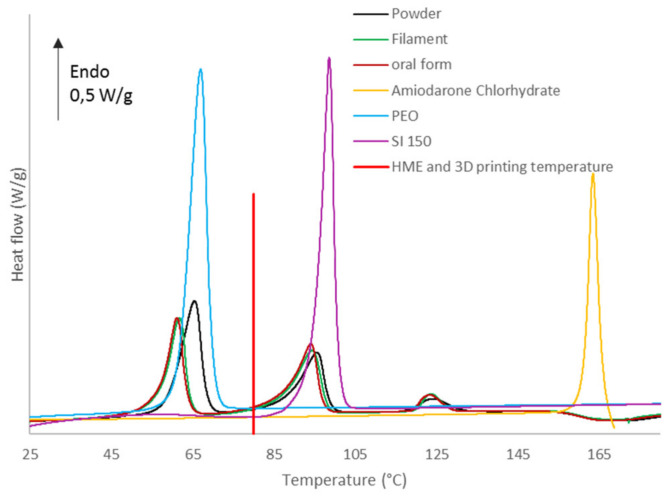
DSC thermoanalytical curves of formulated powder, filament and oral form (formulation B).

Hot melt extrusion and 3D printing heats powder at 80 °C as shown in Figure 12. It can induce change in thermal properties of components such as amorphization. Formulation B contains Merck D-Sorbitol, which is a European Pharmacopeia compliant excipient. This is why it has been analyzed by DSC in powder, filament and oral form as presented in Figure 12. For all samples, three endotherms are observed corresponding respectively to PEO crystalline part melting (around 60–70 °C), D-Sorbitol melting (around 95 °C) and API melting (around 127 °C). Glycerol and silica are present in amounts too low to be identified with DSC analysis. A polymer melting point depression is observed for filament and oral form in comparison to PEO alone. Hot melt extrusion and 3D printing melts the polymer crystalline part which recrystallizes once the material has left the process. This implies a reorganization during recrystallisation inducing a lower intrinsic energy of the PEO matrices. D-Sorbitol and amiodarone hydrochloride present a melting point depression due to interaction with other formulation components [68,69,70]. Considering the extrusion and 3D printing temperature, which is 80 °C, the drug is kept in a crystalline state in the filament and oral forms such as D-Sorbitol. However, a small proportion of D-Sorbitol is melted by the extrusion and 3D printing process. The low proportion of molten D-Sorbitol does not seem to have erased the impact of the particle’s morphology on the impact of the rigidity of the filaments presented previously. D-Sorbitol and API melting are not altered by the HME process as shown by identical endotherm on the thermoanalytical curves between oral form and filament in comparison to formulated powder in Figure 12. Peak area is representative of the product quantity in the formulation. The area of PEO endotherm at 60 °C represents the crystalline quantity of PEO. The crystalline PEO quantity is equivalent between filament and powder with a respective area of 56 J/g and 59 J/g. The powder presents a higher quantity of crystalline PEO with a peak area of 73 J/g. This implies that the initial melting increases the proportion of amorphous PEO.

From the filaments manufactured by HME, oral forms were produced and weighed as presented in Table 8. All filaments produced from different D-Sorbitol grade and suppliers allowed the manufacture of three different dosages demonstrating the capacity of dosage personalization by 3D printing from a filament. Three samples per assay were produced and mass RSD (%) was calculated. Results showed that formulation A allows the production of oral forms with the lowest mass variability identified by an RSD between 0.9% and 1.2%. All the other formulations allowed the manufacture of oral forms with a mass variability greater than 2%. The results showed that the variability in filament diameter impacted the mass variability of the printed oral forms. Indeed, the diameter of the filament conditioned the mass of the latter.

A supplier change can have a big influence on excipient physical properties. So, the use of D-Sorbitol in amounts of 37% by mass can have a big influence on processability or filaments and 3D-printed medicines’ quality [22]. Results shows that sourcing is important in order to choose most suitable excipients to obtain better products quality.

In order to produce precise dose, a calibration on the oral forms masses was made according to the filament length used by the printer. Some teams used the volume of the modeled object as a calibration tool [71]. However, the slicing operation can truncate parts of the volume according to the selected printing settings and therefore induce bias in the mass of the oral forms. That is why the length of the filament was used as a reference value by the printer to make the oral forms. To do so, three objects with different sizes were printed and the software provided a filament length needed. The manufactured objects were weighted, and a calibration curve drawn. The purpose being to link directly the dosage to the filament length.

European Pharmacopoeia 10.3 chapter 2.9.5 indicates that mass uniformity must be carried out on 20 randomly chosen samples. Tablet mass deviation from the average mass has to be smaller than 5 percent for tablets of 250 mg or smaller than 7.5 percent for tablets between 80 mg and 250 mg. A maximum of two tablets can be out of this range and none of them can deviate by more than twice that percentage [24]. Regarding product quantity available in our lab, 10 samples with three different dosages were tested for this study using formulation B because of the pharmaceutical grade of D-Sorbitol SI 150. The mass uniformity study in Table 9 highlights that the three dosages were within the specifications range of the European Pharmacopoeia. An oral form with a theoretical mass of 1000 mg gave 948.63 mg and one with a theorical mass of 750 mg gave 802.53 mg. These two samples were outside the deviation knowing that the European Pharmacopeia authorizes two for 20 samples. It was therefore possible to produce personalized oral forms with an acceptable standard deviation knowing that the variation in filament diameter was the origin of the variation in oral forms masses.

According to the hospital protocol, the oral form disintegration was performed in a syringe containing 5 mL of water under manual agitation. The objective is to disperse the amiodarone in water in order to integrate it into a compote or the content of a baby bottle. It has been observed by Patrojanasophon et al. that the size of the particles present in tablets can influence the disintegration time [72]. This is why the disintegration time will be tested as a function of the D-Sorbitol incorporated in the formulations. As shown in Table 10, all of the oral forms are disaggregated over three minutes in 5 mL of water. It has therefore been possible to produce oral forms of rapidly disintegrating amiodarone which can be dispersed in water. Whatever D-Sorbitol used, the disintegration time was unchanged. Particle size and filament diameter variability did not influence the release performance of the oral forms.

## 4. Conclusions

Formulations to treat arrhythmia in children were manufactured by using D-Sorbitol from several suppliers. By the HME process, filaments were produced in order to 3D print personalized oral forms. Whatever D-Sorbitol origin, all filaments can be used despite their different particle’s properties.

Newly studied, the filling agent properties influence the hot melt extrusion process and the filament characteristics. One of the essential filament characteristics, to obtain oral forms with a reproducible mass, is the diameter variability. Carlo Erba’s D-Sorbitol is the product with the weakest flowability, mainly because of its large quantity of particles smaller than 45 microns. Despite this weakness, filament production occurs with a better constancy of flow and therefore a better reproducibility on the filament diameter compared to formulations using other D-Sorbitol Suppliers.

Three-point bending analysis showed that D-Sorbitol particles size have no impact on the filament mechanical properties, but particles’ morphologies can impact filament stiffness.

Using the filament produced by HME and an innovating calibration method based on the filament length, it was possible to carry out three dosages of 125 mg, 750 mg and 1000 mg with acceptable mass uniformity. This formulation will be tested within a pediatric unit in a public hospital. This opens new ways of producing oral forms adapted to the patient’s needs.

## Figures and Tables

**Table 1:** Summary table of the percentage of each product in the formulation.

Product	Mass Percentage (%)
PEO	40%
D-Sorbitol	37%
Glycerol	2%
SiO_2_	1%
Amiodarone Hydrochloride	20%

**Table 2:** Summary of D-Sorbitol used for the preparation of formulation A, A^T^, B, C and D.

Formulation	A	A^T^	B	C	D
D-Sorbitol used	CE	CE^T^	SI150	SI200	GLT

**Table 3:** Three-dimensional printing slicing settings.

Parameter	Value
Layer heights	0.4 mm
Nozzle temperature	80 °C
Bed temperature	30 °C
Number of top and bottom layers	0
Number of perimeters	0
Infill	40%
Infill pattern	Rectilinear
Speed for print moves	10 mm/s
Speed for non-print moves	120 mm/s

**Table 4:** Summary of thermal analysis.

D-Sorbitol	CE	CE^T^	SI150	SI200	GLT
Onset (°C)	97.9 ± 0.12	97.9 ± 0.05	96.1 ± 0.17	95.7 ± 0.14	98.4 ± 0.03
Water loss (% weight)	0.4 ± 0.15	0.3 ± 0.06	0.3 ± 0.06	0.4 ± 0.07	0.3 ± 0.06

**Table 5:** Summary of D-Sorbitol CE, CE^T^, SI150, SI200 and GLT flowability measurement (green for the best flowability results and red for the worst flowability results).

D-Sorbitol Used	CE	CE^T^	SI150	SI200	GLT
Carr’s index	24.2	22	13.8	19	11
Hausner ratio	1.2	1.1	1.2	1.1	1.1
Angle of repose (°)	30.2	23.1	23.6	25	26.4

**Table 6:** Results of filament diameter by stericut measurement from left to right formulation A [41], A^T^, B, C and D.

Formulation	A	A^T^	B	C	D
Average diameter (mm)	1.701	1.697	1.701	1.701	1.702
Diameter Relative Standard Deviation (RSD) (%)	1.5	2.0	2.9	2.5	2.4
Regulation belt speed average (mm/s)	6.9	6.7	6.5	6.5	6.1

**Table 7:** Summary results of three-point bending.

Formulation	Elastic Distance (mm)	Stiffness (N/mm)
A	1.06 ± 0.08	1.19 ± 0.07
A^T^	1.32 ± 0.17	1.02 ± 0.05
B	1.10 ± 0.08	2.03 ± 0.10
C	1.10 ± 0.00	2.08 ± 0.17
D	1.31 ± 0.10	2.23 ± 0.08

**Table 8:** Summary of oral forms masses printed from formulation A, A^T^, B, C and D.

Formulation	Average Weighed Mass (mg) (N = 3)	Mass RSD (%)
A	111.95	1.21%
	776.17	1.11%
	982.58	0.94%
A^T^	108.50	2.65%
	724.00	2.98%
	962.42	2.29%
B	136.61	6.07%
	745.29	4.64%
	996.73	3.45%
C	112.74	2.06%
	702.54	3.35%
	968.41	6.75%
D	114.35	3.43%
	715.56	2.96%
	964.29	2.58%

**Table 9:** Mass uniformity of oral forms produced with formulation B. In red are indicated the oral forms coming out of the 5% mass deviation. All batches comply with the specifications of the European Pharmacopoeia.

Theorical Mass (mg)	Filament Length Used (mm)	Weighed Mass (mg)					Average (mg)	Mass RSD (%)
1000	401.6	1032.96	1018.45	1005.11	974.72	983.49	996.17	2.9%
		985.30	1043.92	985.17	983.90	948.63		
750	301.2	774.78	733.52	734.92	802.53	767.21	754.79	2.9%
		741.36	744.72	751.56	738.84	758.41		
125	50.2	129.63	127.16	120.70	124.24	125.70	125.71	3.4%
		132.75	123.83	123.57	119.07	130.47		

**Table 10:** Disintegration time on three oral form samples produced by A, A^T^, B, C and D batches.

	A	A^T^	B	C	D
Average mass (mg)	112	121	122	113	114
Disaggregation time (min)	3	3	3	3	3

## Data Availability

Not applicable.

## References

[1] Lazarou J., Pomeranz B.H., Corey P.N. (1998). Incidence of adverse drug reactions in hospitalized patients: A meta-analysis of prospective studies. JAMA.

[2] Cohen J.S. (1999). Ways to minimize adverse drug reactions. individualized doses and common sense are key. Postgrad. Med..

[3] Vijayavenkataraman S., Fuh J.Y.H., Lu W.F. (2017). 3D printing and 3D bioprinting in pediatrics. Bioengineering.

[4] Ali A., Ahmad U., Akhtar J. (2020). 3D printing in pharmaceutical sector: An overview. Pharm. Formul. Des. Recent Pract..

[5] Kapetaniou C., Rieple A., Pilkington A., Frandsen T., Pisano P. (2018). Building the layers of a new manufacturing taxonomy: How 3D printing is creating a new landscape of production eco-systems and competitive dynamics. Technol. Forecast. Soc. Chang..

[6] Cailleaux S., Sanchez-Ballester N.M., Gueche Y.A., Bataille B., Soulairol I. (2021). Fused Deposition Modeling (FDM), the new asset for the production of tailored medicines. J. Control. Release.

[7] Lee J.-Y., An J., Chua C.K. (2017). Fundamentals and applications of 3D printing for novel materials. Appl. Mater. Today.

[8] Melocchi A., Parietti F., Maroni A., Foppoli A., Gazzaniga A., Zema L. (2016). Hot-melt extruded filaments based on pharmaceutical grade polymers for 3D printing by fused deposition modeling. Int. J. Pharm..

[9] Öblom H., Sjöholm E., Rautamo M., Sandler N. (2019). Towards printed pediatric medicines in hospital pharmacies: Comparison of 2D and 3D-printed orodispersible warfarin films with conventional oral powders in unit dose sachets. Pharmaceutics.

[10] Awad A., Trenfield S.J., Goyanes A., Gaisford S., Basit A.W. (2018). Reshaping drug development using 3D printing. Drug Discov. Today.

[11] Merck Working with EOS’ AMCM to Produce Next-Generation 3D Printed Tablets. https://3dprintingindustry.com/news/merck-working-with-eos-amcm-to-produce-next-generation-3d-printed-tablets-168729.

[12] Kempin W., Domsta V., Grathoff G., Brecht I., Semmling B., Tillmann S., Weitschies W., Seidlitz A. (2018). Immediate release 3D-printed tablets produced via fused deposition modeling of a thermo-sensitive drug. Pharm. Res..

[13] Goyanes A., Kobayashi M., Martínez-Pacheco R., Gaisford S., Basit A.W. (2016). Fused-filament 3D printing of drug products: Microstructure analysis and drug release characteristics of PVA-based caplets. Int. J. Pharm..

[14] Yang Y., Wang H., Li H., Ou Z., Yang G. (2018). 3D printed tablets with internal scaffold structure using ethyl cellulose to achieve sustained ibuprofen release. Eur. J. Pharm. Sci..

[15] Gioumouxouzis C.I., Baklavaridis A., Katsamenis O.L., Markopoulou C.K., Bouropoulos N., Tzetzis D., Fatouros D.G. (2018). A 3D printed bilayer oral solid dosage form combining metformin for prolonged and glimepiride for immediate drug delivery. Eur. J. Pharm. Sci..

[16] European Commission (2015). Official Journal C 95/2015.

[17] Tan D.K., Maniruzzaman M., Nokhodchi A. (2018). Advanced pharmaceutical applications of hot-melt extrusion coupled with fused deposition modelling (FDM) 3D printing for personalised drug delivery. Pharmaceutics.

[18] Saviano M., Aquino R.P., Del Gaudio P., Sansone F., Russo P. (2019). Poly(Vinyl Alcohol) 3D printed tablets: The effect of polymer particle size on drug loading and process efficiency. Int. J. Pharm..

[19] Shaqour B., Samaro A., Verleije B., Beyers K., Vervaet C., Cos P. (2020). Production of drug delivery systems using fused filament fabrication: A systematic review. Pharmaceutics.

[20] Gültekin H.E., Tort S., Acartürk F. (2019). An effective technology for the development of immediate release solid dosage forms containing low-dose drug: Fused deposition modeling 3D printing. Pharm. Res..

[21] Jani R., Patel D. (2015). Hot melt extrusion: An industrially feasible approach for casting orodispersible film. Asian J. Pharm. Sci..

[22] Öblom H., Zhang J., Pimparade M., Speer I., Preis M., Repka M., Sandler N. (2019). 3D-printed isoniazid tablets for the treatment and prevention of tuberculosis—Personalized dosing and drug release. AAPS Pharmscitech.

[23] Okwuosa T.C., Stefaniak D., Arafat B., Isreb A., Wan K.-W., Alhnan M.A. (2016). A lower temperature FDM 3D printing for the manufacture of patient-specific immediate release tablets. Pharm. Res..

[24] Pietrzak K., Isreb A., Alhnan M.A. (2015). A flexible-dose dispenser for immediate and extended release 3D printed tablets. Eur. J. Pharm. Biopharm..

[25] Sadia M., Arafat B., Ahmed W., Forbes R.T., Alhnan M.A. (2018). Channelled tablets: An innovative approach to accelerating drug release from 3D printed tablets. J. Control. Release.

[26] Aho J., Bøtker J.P., Genina N., Edinger M., Arnfast L., Rantanen J. (2019). Roadmap to 3D-printed oral pharmaceutical dosage forms: Feedstock filament properties and characterization for fused deposition modeling. J. Pharm. Sci..

[27] Desai D., Sandhu H., Shah N., Malick W., Zia H., Phuapradit W., Vaka S.R.K. (2018). Selection of solid-state plasticizers as processing aids for hot-melt extrusion. J. Pharm. Sci..

[28] Moseson D.E., Taylor L.S. (2018). The application of temperature-composition phase diagrams for hot melt extrusion processing of amorphous solid dispersions to prevent residual crystallinity. Int. J. Pharm..

[29] Pereira B.C., Isreb A., Forbes R.T., Dores F., Habashy R., Petit J.-B., Alhnan M.A., Oga E.F. (2019). ‘Temporary Plasticiser’: A novel solution to fabricate 3D printed patient-centred cardiovascular ‘polypill’ architectures. Eur. J. Pharm. Biopharm..

[30] Tian H., Liu D., Yao Y., Ma S., Zhang X., Xiang A. (2017). Effect of sorbitol plasticizer on the structure and properties of melt processed polyvinyl alcohol films. J. Food Sci..

[31] Pawar H.V., Tetteh J., Boateng J.S. (2013). Preparation, optimisation and characterisation of novel wound healing film dressings loaded with streptomycin and diclofenac. Colloids Surf. B Biointerfaces.

[32] Alhijjaj M., Belton P., Qi S. (2016). An investigation into the use of polymer blends to improve the printability of and regulate drug release from pharmaceutical solid dispersions prepared via fused deposition modeling (FDM) 3D printing. Eur. J. Pharm. Biopharm..

[33] Melocchi A., Loreti G., Del Curto M.D., Maroni A., Gazzaniga A., Zema L. (2015). Evaluation of hot-melt extrusion and injection molding for continuous manufacturing of immediate-release tablets. J. Pharm. Sci..

[34] Salehi S., Boddohi S. (2017). New formulation and approach for mucoadhesive buccal film of rizatriptan benzoate. Prog. Biomater..

[35] Pagliaro M., Rossi M. (2008). The Future of Glycerol: New Uses of a Versatile Raw Material.

[36] Karl M., Nalawade S., Com A., Djuric D., Kolter K. Suitability of pure and plasticized polymers for hot melt extrusion. Proceedings of the The 37th Annual Meeting and Exposition of the Controlled Release Society.

[37] Tran D.T., Majerová D., Veselý M., Kulaviak L., Ruzicka M.C., Zámostný P. (2019). On the mechanism of colloidal silica action to improve flow properties of pharmaceutical excipients. Int. J. Pharm..

[38] Augsburger L.L., Shangraw R.F. (1966). Effect of glidants in tableting. J. Pharm. Sci..

[39] Marraffa J.M., Wexler P. (2014). Amiodarone. Encyclopedia of Toxicology.

[40] Guillory J.K. (2003). Handbook of aqueous solubility data by Samuel H. Yalkowsky and Yan He; CRC Press: Boca Raton, FL. 2003. Xii + 1496 Pp. 18 × 26 Cm. ISBN 0-89493-1532-8. $299.95. J. Med. Chem..

[41] Roulon S., Soulairol I., Lavastre V., Payre N., Cazes M., Delbreilh L., Alié J. (2021). Production of reproducible filament batches for the fabrication of 3D printed oral forms. Pharmaceutics.

[42] Donnelly R.F., Majithiya R., Singh T.R.R., Morrow D.I.J., Garland M.J., Demir Y.K., Migalska K., Ryan E., Gillen D., Scott C.J. (2011). Design, optimization and characterisation of polymeric microneedle arrays prepared by a novel laser-based micromoulding technique. Pharm. Res..

[43] Goyanes A., Chang H., Sedough D., Hatton G.B., Wang J., Buanz A., Gaisford S., Basit A.W. (2015). Fabrication of controlled-release budesonide tablets via desktop (FDM) 3D printing. Int. J. Pharm..

[44] Council of Europe (2010). 2.9.36. Powder Flow—European Pharmacopoeia 10.3.

[45] Council of Europe (2019). 2.9.34. Bulk Density and Tappe—European Pharmacopoeia 10.3.

[46] Chattoraj S., Sun C.C. (2018). Crystal and particle engineering strategies for improving powder compression and flow properties to enable continuous tablet manufacturing by direct compression. J. Pharm. Sci..

[47] Juarez-Enriquez E., Olivas G.I., Zamudio-Flores P.B., Ortega-Rivas E., Perez-Vega S., Sepulveda D.R. (2017). Effect of water content on the flowability of hygroscopic powders. J. Food Eng..

[48] Sandler N., Reiche K., Heinämäki J., Yliruusi J. (2010). Effect of moisture on powder flow properties of theophylline. Pharmaceutics.

[49] Afoakwa E.O., Paterson A., Fowler M., Vieira J. (2008). Characterization of melting properties in dark chocolates from varying particle size distribution and composition using differential scanning calorimetry. Food Res. Int..

[50] Rosa F., Corvis Y., Lai-Kuen R., Charrueau C., Espeau P. (2015). Influence of particle size on the melting characteristics of organic compounds. J. Therm. Anal. Calorim..

[51] Conceição J., Estanqueiro M., Amaral M.H., Silva J.P.S., Lobo J.M. (2014). Technological excipients of tablets: Study of flow properties and compaction behavior. AJMSM.

[52] Geldart D., Abdullah E.C., Hassanpour A., Nwoke L.C., Wouters I. (2006). Characterization of powder flowability using measurement of angle of repose. China Particuology.

[53] Osorio J.G., Muzzio F.J. (2013). Effects of powder flow properties on capsule filling weight uniformity. Drug Dev. Ind. Pharm..

[54] Stavrou A.G., Hare C., Hassanpour A., Wu C.-Y. (2020). Investigation of powder flowability at low stresses: Influence of particle size and size distribution. Powder Technol..

[55] Jiang Y.-J., Fan X.-Y., Li T.-H., Xiao S.-Y. (2018). Influence of particle-size segregation on the impact of dry granular flow. Powder Technol..

[56] Gilbertson M.A., Eames I. (2003). the influence of particle size on the flow of fluidised powders. Powder Technol..

[57] Koopmans R., Doelder J.D., Molenaar J., Doelder J.D., Molenaar J. (2010). Polymer Melt Fracture.

[58] Lin Y.-H. (1985). Explanation for slip-stick melt fracture in terms of molecular dynamics in polymer melts. J. Rheol..

[59] Yamamoto M. (2019). Elucidation of mechanisms for stick slip melt fracture of polymer melt (experimental analysis of unstable flow of high density polyethylene melt). Trans. JSME.

[60] Baldi F., Briatico-Vangosa F., Franceschini A. (2014). Experimental study of the melt fracture behavior of filled high-density polyethylene melts. Polym. Eng. Sci..

[61] Hristov V., Vlachopoulos J. (2008). Effects of polymer molecular weight and filler particle size on flow behavior of wood polymer composites. Polym. Compos..

[62] Patil H., Tiwari R.V., Repka M.A. (2015). Hot-melt extrusion: From theory to application in pharmaceutical formulation. AAPS Pharmscitech.

[63] Nasereddin J.M., Wellner N., Alhijjaj M., Belton P., Qi S. (2018). Development of a simple mechanical screening method for predicting the feedability of a pharmaceutical FDM 3D printing filament. Pharm. Res..

[64] Prasad E., Islam M.T., Goodwin D.J., Megarry A.J., Halbert G.W., Florence A.J., Robertson J. (2019). Development of a hot-melt extrusion (HME) process to produce drug loaded affinisol^TM^ 15lv filaments for fused filament fabrication (FFF) 3D printing. Addit. Manuf..

[65] Zhang J., Feng X., Patil H., Tiwari R.V., Repka M.A. (2017). Coupling 3D printing with hot-melt extrusion to produce controlled-release tablets. Int. J. Pharm..

[66] Fu S.-Y., Feng X.-Q., Lauke B., Mai Y.-W. (2008). Effects of particle size, particle/matrix interface adhesion and particle loading on mechanical properties of particulate—Polymer composites. Compos. Part. B Eng..

[67] Ji X.L., Jing J.K., Jiang W., Jiang B.Z. (2002). Tensile modulus of polymer nanocomposites. Polym. Eng. Sci..

[68] Meng F., Trivino A., Prasad D., Chauhan H. (2015). Investigation and correlation of drug polymer miscibility and molecular interactions by various approaches for the preparation of amorphous solid dispersions. Eur. J. Pharm. Sci..

[69] Schammé B., Couvrat N., Tognetti V., Delbreilh L., Dupray V., Dargent É., Coquerel G. (2018). Investigation of drug–excipient interactions in biclotymol amorphous solid dispersions. Mol. Pharm..

[70] Schammé B., Couvrat N., Malpeli P., Dudognon E., Delbreilh L., Dupray V., Dargent É., Coquerel G. (2016). Transformation of an active pharmaceutical ingredient upon high-energy milling: A process-induced disorder in biclotymol. Int. J. Pharm..

[71] EDQM 2.9.5. Uniformity of Mass of Single Dose Preparation—European Pharmacopoeia 10.2. https://pheur.edqm.eu/app/10-2/content/10-2/20905E.htm?highlight=on&terms=uniformity&terms=mass.

[72] Patrojanasophon P., Ngawhirunpat T., Akkaramongkolporn P., Opanasopit P., Nattapulwat N. (2017). Effect of particle size and diluent type on critical parameters for disintegration of tablets containing croscarmellose sodium as a disintegrant. Trop. J. Pharm. Res..

